# Seasonal Pattern and Age-Specific Detection of Eight Respiratory Viruses Causing Acute Respiratory Infection in 2024, Bangkok, Thailand

**DOI:** 10.3390/tropicalmed10120339

**Published:** 2025-11-29

**Authors:** Nungruthai Suntronwong, Preeyaporn Vichaiwattana, Jiratchaya Puenpa, Siripat Pasittungkul, Ratchadawan Aeemjinda, Lakkhana Wongsrisang, Yong Poovorawan

**Affiliations:** 1 Center of Excellence in Clinical Virology, Faculty of Medicine, Chulalongkorn University, Bangkok 10330, Thailand; nungruthai.s@chula.ac.th (N.S.); preeyaporn.vic@chulahospital.org (P.V.); jiratchaya.pu@gmail.com (J.P.); spsiripat@hotmail.com (S.P.); aeemjinda.r@gmail.com (R.A.); lakkhana4118@gmail.com (L.W.); 2 The Royal Society of Thailand (FRS(T)), Sanam Sueapa, Dusit, Bangkok 10330, Thailand

**Keywords:** epidemiology, acute respiratory infections, seasonal, virus

## Abstract

Since the emergence of COVID-19, the epidemiological and seasonal patterns of respiratory pathogens have shifted, highlighting the need for ongoing surveillance. This study investigated the epidemiology, seasonal trends, and age-specific detection of respiratory viruses among patients with acute respiratory infections (ARIs) in Thailand from January to December 2024. Eight respiratory viruses were detected using multiplex real-time RT-PCR. Of 7853 samples, 60.8% (4777) tested positive. The most frequently detected pathogens were influenza virus (IFV, 24.8%), SARS-CoV-2 (21.5%), and human rhinovirus (HRV, 20.8%). IFV showed biannual peaks during the cold and rainy seasons, SARS-CoV-2 peaked in the warm months, and HRV circulated year-round. Respiratory syncytial virus (RSV) and human metapneumovirus (HMPV) were primarily detected during the rainy season (July–November), reflecting a return toward pre–COVID-19 seasonal patterns. Age-specific differences were notable: HRV was most prevalent in children < 5 years, IFV predominated among those aged 6–18 years, and adults ≥ 19 years were mainly positive for IFV and SARS-CoV-2. Co-infections were most frequent in children aged 3–5 years, often involving HRV. These findings provide updated insights into post–COVID-19 viral epidemiology, emphasize the importance of age- and season-specific surveillance, and support the development of effective public health strategies for ARI control.

## 1. Introduction

Acute respiratory infections (ARIs), encompassing both upper and lower respiratory tract infections, are associated with high morbidity and mortality [1]. Globally, viral pathogens represent the second and third most common contributors to respiratory infection episodes, accounting for an estimated 46.4 million cases [2]. A wide range of viruses are implicated, including influenza viruses (IFV), respiratory syncytial virus (RSV), human rhinovirus (HRV), human metapneumovirus (HMPV), human parainfluenza virus (HPIV), human coronaviruses (HCoV), severe acute respiratory syndrome coronavirus 2 (SARS-CoV-2), and adenoviruses (HAdV), which are among the most frequently detected in patients with acute respiratory disease [3]. The circulation of these pathogens is influenced by multiple factors, including seasonality, environmental conditions, geographic location, waning infection- or vaccine-induced immunity, patient age, and human behavior [4,5,6].

Most respiratory viruses exhibit distinct seasonal circulation patterns across climate zones. In temperate regions, annual winter peaks are common, whereas tropical regions display either region-specific peaks or year-round activity [4,7,8]. However, the COVID-19 pandemic substantially disrupted these established seasonal trends, primarily due to the widespread implementation of non-pharmaceutical interventions (NPIs), such as mask-wearing, social distancing, and lockdowns, which markedly reduced respiratory virus circulation [7,9,10]. Following the relaxation of NPIs, a resurgence of IFV, RSV, and other respiratory viruses has been reported in several countries, particularly in temperate regions, often accompanied by shifts in seasonality and delayed peak timings compared with pre–pandemic patterns [8,9,11,12]. These resurgences underscore the importance of continuous surveillance of ARIs and their viral etiologies. Moreover, understanding the circulation of SARS-CoV-2 alongside other respiratory pathogens remains critical for public health planning.

Unlike temperate regions, Thailand is situated in the tropics, where respiratory viruses display unique circulation dynamics, with each virus exhibiting a distinct seasonal pattern [13]. Following the COVID-19 pandemic, a clear rebound of respiratory viral infections was observed in Thailand [14], highlighting the importance of ongoing monitoring of respiratory pathogens to guide effective public health strategies. In this study, we investigated eight viral respiratory pathogens using multiplex real-time RT-PCR in patients of all ages with ARIs and characterized their seasonal distribution between January and December 2024 in Bangkok, Thailand. Our findings demonstrate seasonal dynamics and age-specific viral detection patterns during the post–COVID-19 era, providing critical insights to inform public health preparedness for future respiratory epidemics.

## 2. Materials and Methods

### 2.1. Study Design, Sample Collection and Ethical Approvals

We conducted a retrospective cohort study in Bangkok, Thailand, from January to December 2024. Patients presenting with ARI, defined as a temperature ≥ 38 °C with cough and symptom onset within the preceding 10 days, who visited Bangpakok 9 Hospital, were enrolled. Nasopharyngeal samples were collected, placed in 1 mL of universal transport medium, and sent to the Center of Excellence in Clinical Virology, King Chulalongkorn Memorial Hospital, for respiratory virus testing as part of routine surveillance. Samples collected from the same individual within ≤4 weeks or during follow-up visits were excluded.

The study protocol was approved by the Institutional Review Board of the Faculty of Medicine, Chulalongkorn University (IRB No. 0977/67) and conducted in accordance with the Declaration of Helsinki and Good Clinical Practice guidelines. All demographic data and patient identifiers were anonymized to ensure confidentiality. Owing to the retrospective nature of the study, the requirement for informed consent was waived by the IRB (IRB No. 0977/67). Permission to use the study data was granted by the director of Bangpakok 9 International Hospital.

### 2.2. Real-Time RT-PCR

Viral nucleic acids were extracted from 200 µL of clinical samples using the magLEAD 12gC system (Precision System Science, Chiba, Japan), following the manufacturer’s instructions. Extracts were eluted in 50 µL of buffer and tested for respiratory viruses using real-time RT-PCR, as previously described [15,16,17,18,19,20,21]. Primer and probe sequences are summarized in Supplementary Table S1. Targets included: the matrix (M) gene for IFV and RSV, fusion (F) gene for HMPV, 5′ untranslated region for HRV, hemagglutinin-neuraminidase gene for HPIV, hexon gene for HAdV, ORF1ab gene for HCoV, and nucleocapsid (N1 and N2) genes for SARS-CoV-2.

For RNA viruses, RT-PCR was performed using the SensiFAST Probe No-ROX One-Step Kit (Bioline, London, UK) according to the manufacturer’s instructions. Each 10 µL reaction contained 2 µL of RNA (100 ng^–1^ µg), 5 µL of 2× SensiFAST Probe No-ROX Mix, 0.4 µL each of forward and reverse primers (10 µM), 0.1 µL of probe, 0.2 µL of RNase inhibitor, 0.1 µL of reverse transcriptase, and nuclease-free water. Thermal cycling conditions were reverse transcription at 42 °C for 20 min, initial denaturation at 95 °C for 3 min, followed by 45 amplification cycles of 95 °C for 10 s and 60 °C for 20 s. For DNA viruses, the same reagent mix was used, except that each 10 µL reaction contained 2 µL of DNA instead of RNA and no reverse transcriptase. Cycling conditions were initial denaturation at 95 °C for 3 min, followed by 45 cycles of 95 °C for 10 s and 60 °C for 20 s. Each run included positive and negative controls, along with amplification of the glyceraldehyde-3-phosphate dehydrogenase (GAPDH) gene as an internal control. Samples were considered positive if the cycle threshold (Ct) value was ≤38.

### 2.3. Statistical Analysis

Participants were stratified into six age groups: infants (0–2 years), preschool children (3–5 years), school-aged children (6–12 years), adolescents (13–18 years), adults (19–60 years), and older adults (>60 years). The positivity rate was calculated as the proportion of positive samples relative to the total number of samples collected. The co-infection rate was determined when samples tested positive for more than two pathogens and was defined as the number of positive samples with co-infection divided by the total number of samples tested. Seasonal periods were defined as follows: warm season (March–May), rainy season (June–October), and cold season (November–February). Differences in positivity rates across age groups were assessed using the chi-square test or Fisher’s exact test, as appropriate. Figures were generated using R software version 4.5.1 (R Foundation for Statistical Computing, Vienna, Austria). Statistical analyses were performed using IBM SPSS Statistics v23.0 (IBM Corp., Armonk, NY, USA). A *p*-value < 0.05 was considered statistically significant.

## 3. Results

### 3.1. Overall Prevalence of Respiratory Viruses and Co-Infection Patterns

**Figure 1:**
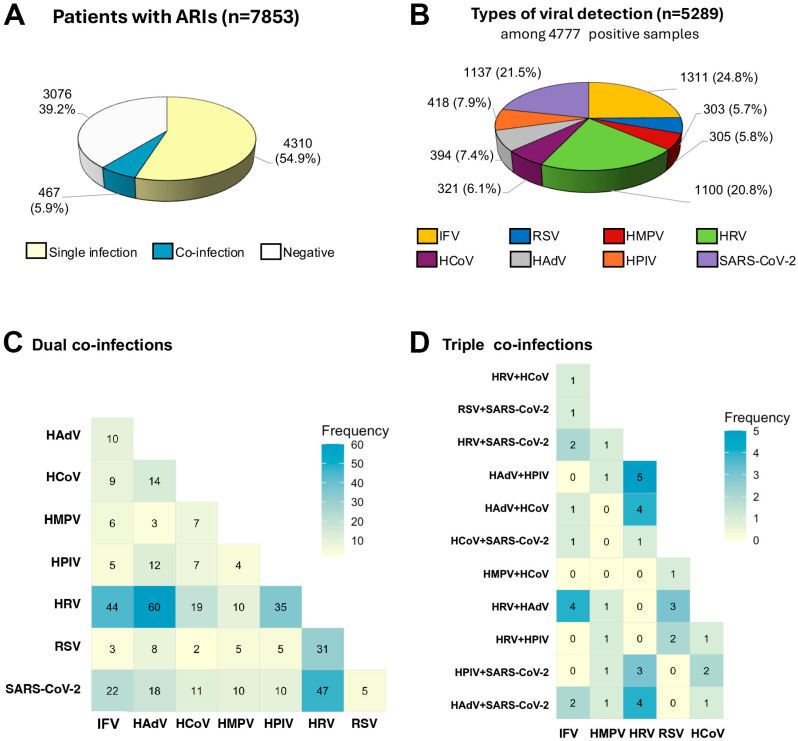
Types of viral detection and co-infection patterns. (**A**) Proportion of patients with ARIs (n = 7853) who tested negative or positive for viral infections, including single and co-infections. (**B**) Distribution of detected viral pathogens (n = 5289) among 4777 infected patients. Co-infection patterns detected among eight viral pathogens are shown as (**C**) dual and (**D**) triple co-infections. Color intensity correspond to the frequency of positive sample detection.

Between January and December 2024, a total of 7853 consecutive respiratory samples were collected from patients with ARI. Of these patients, 54.9% were female, and the mean age was 28.1 years (SD, 22.5; range, 1 month to 98 years) (Table 1). The study population included 705 infants (0–2 years; 9.0%), 971 preschool children (3–5 years; 12.3%), 1228 school-aged children (6–12 years; 15.6%), 399 adolescents (13–18 years; 5.1%), 3789 adults (19–60 years; 48.3%), and 761 older adults (>60 years; 9.7%). All samples were retrospectively tested for eight viral pathogens. Overall, 60.8% (4777/7853) of samples tested positive for at least one respiratory virus, including 54.9% (4310/7853) with single infections and 5.9% (467/7853) with co-infections (Figure 1A). There were 5289 positive pathogens detected among 4777 infected individuals. IFV was the most frequently detected pathogen (24.8%, 1311/5289), followed by SARS-CoV-2 (21.5%, 1137/5289), HRV (20.8%, 1100/5289), HPIV (7.9%, 418/5289), HAdV (7.4%, 394/5289), HCoV (6.1%, 321/5289), HMPV (5.8%, 305/5289), and RSV (5.7%, 303/5289) (Figure 1B). The most common dual co-infections were HRV + HAdV, HRV + SARS-CoV-2, and HRV + IFV (Figure 1C). The most frequent triple co-infections were HRV + HAdV + HPIV, HRV + HAdV + HCoV, and either HRV + HAdV + IFV or HRV + HAdV + SARS-CoV-2 (Figure 1D).

### 3.2. Seasonal Distribution of Viral Prevalence

**Figure 2:**
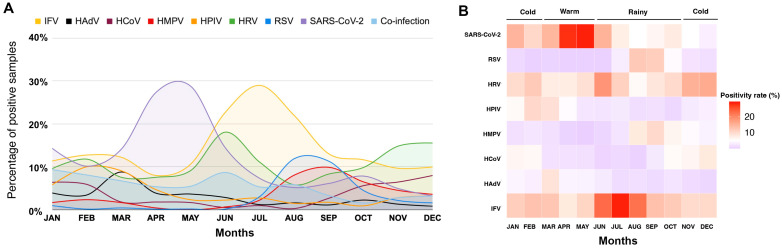
Monthly distribution of viral pathogens detected in patients with ARI (n = 7853) in Bangkok, Thailand, from January to December 2024. (**A**) Monthly positivity rates of viral pathogen detection, displayed in different colors. (**B**) Heat map of viral activity corresponding to seasonal patterns: cold months (November–February), warm months (March–May), and rainy months (June–October).

To examine the seasonal distribution of viral pathogens, we analyzed the monthly prevalence of positive samples for each virus. Influenza activity exhibited a minor peak during January–March (cool season), with positivity rates ranging from 11.3% to 12.7%, and a major peak during June–August (rainy season), with rates between 21.9% and 29.0% (Figure 2A,B). SARS-CoV-2 peaked in April–May (warm season), with positivity rates of 27.3–28.8%. HRV circulated year-round, showing dual peaks in June–July (11–18%) and November–December (14.8–15.5%). HPIV, HCoV, and HAdV were more commonly detected during the cooler months, although at relatively low prevalence. RSV activity was observed between July and October (rainy season), with a similar pattern also noted for HMPV.

### 3.3. Age-Associated Viral Pathogen Detection

**Figure 3:**
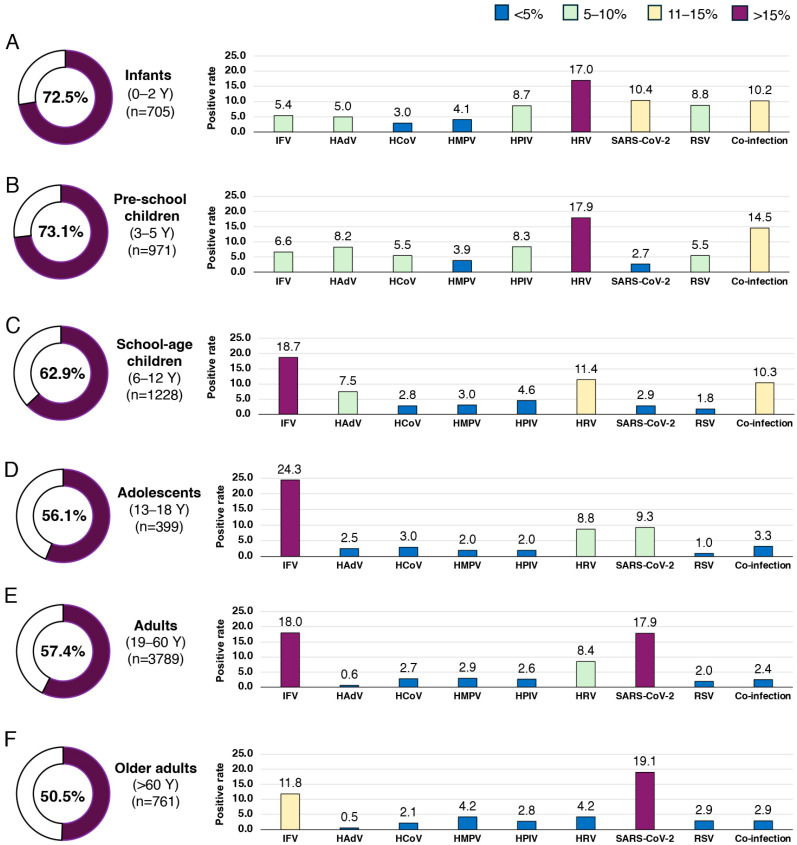
Positive rates and viral pathogen distribution by age group. Pie charts show the positivity rates for (**A**) infants, (**B**) pre-school children, (**C**) school-age children, (**D**) adolescents, (**E**) adults, and (**F**) older adults. All eight viral pathogens, including influenza virus (IFV), human adenovirus (HAdV), seasonal human coronavirus (HCoV), human metapneumovirus (HMPV), human parainfluenza virus (HPIV), human rhinovirus (HRV), severe acute respiratory syndrome coronavirus 2 (SARS-CoV-2), respiratory syncytial virus (RSV) as well as co-infections, were tested. Numbers above the bars indicate the positivity rate for each virus.

**Figure 4:**
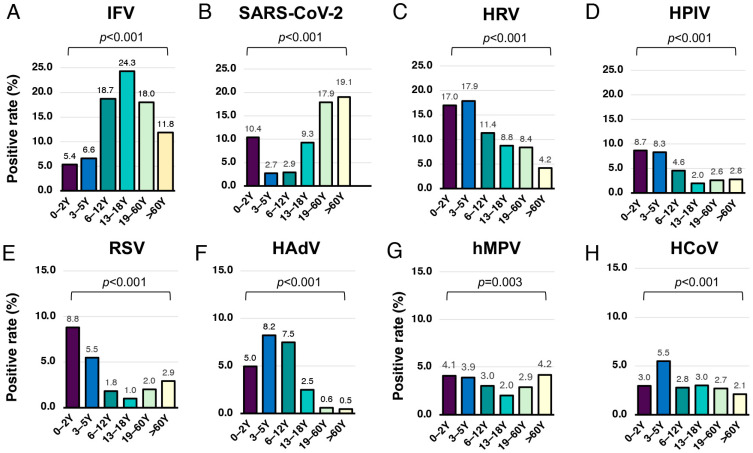
Comparison of positivity rates among age groups. All eight viral pathogens, including (**A**) influenza virus (IFV), (**B**) severe acute respiratory syndrome coronavirus 2 (SARS-CoV-2), (**C**) human rhinovirus (HRV), (**D**) human parainfluenza virus (HPIV), (**E**) respiratory syncytial virus (RSV), (**F**) human adenovirus (HAdV), (**G**) human metapneumovirus (HMPV) and (**H**) seasonal human coronavirus (HCoV) were tested. Positivity rates for each viral pathogen were compared across six age groups using the chi-square test. Numbers above the bars indicate the positivity rate for each virus. A *p*-value < 0.05 was considered statistically significant.

To evaluate age-specific viral pathogen detection, samples were stratified into six age groups: infants (0–2 years), preschool children (3–5 years), school-aged children (6–12 years), adolescents (13–18 years), adults (19–60 years), and older adults (>60 years). The overall positivity rate was 72.5% (511/705) among infants, with HRV, SARS-CoV-2, and RSV being the most frequently detected pathogens (Figure 3A). Among preschool children, 73.1% (710/971) tested positive, with HRV predominating, followed by HPIV and HAdV (Figure 3B). In school-aged children, the positivity rate was 62.9% (773/1228), with IFV as the most common pathogen, followed by HRV and HAdV (Figure 3C). Among adolescents (13–18 years) and adults (19–60 years), positivity rates were 56.1% (224/399) and 57.4% (2175/3789), respectively (Figure 3D,E). In both groups, IFV, SARS-CoV-2, and HRV were the three most frequently detected pathogens. In older adults (>60 years), 50.5% (384/761) tested positive, with SARS-CoV-2 and IFV being the most common (Figure 3F). Overall, the positivity rate of viral detection differed significantly across age groups (Figure 4).

## 4. Discussion

In this study, we investigated the positivity rates of eight respiratory viruses in Thailand from January to December 2024. Our analysis revealed distinct monthly activity patterns for these viruses, as well as age-specific variations in viral detection. Overall, the eight viruses collectively accounted for 60.8% of all-age patients with ARIs. The three most prevalent pathogens were IFV, SARS-CoV-2, and HRV, consistent with global respiratory infection trends [22]. Viral positivity was higher in children than in adults and the elderly, with each age group showing a distinct predominance of specific viruses. Consistent with a previous study [8,23], the viral–viral co-infection rate among all age groups was less than 10% and, co-infections were most frequent in children, particularly those involving HRV and HAdV in combination with other viruses.

Compared with our previous study published in 2023 [14], the novelty of the present work lies in its extended surveillance period through 2024, when respiratory virus circulation had largely stabilized in the post–pandemic phase. This enabled a more accurate characterization and prediction of the seasonal patterns of respiratory viruses, which may not have been fully established in the earlier study [14]. Furthermore, the current study included a larger sample size and utilized individual real-time RT-PCR assays for each virus instead of a commercial multiplex panel, thereby enhancing the sensitivity and specificity of viral detection [15,16,17,18,19,20,21].

The sequential seasonal peaks of respiratory viruses in Thailand during 2024 were characterized by an initial high-intensity peak of SARS-CoV-2, followed by successive peaks of IFV, HRV, RSV, and HMPV. Other respiratory viruses circulated at low prevalence without a clear seasonal pattern. Overall, the observed seasonality more closely resembled pre–pandemic patterns, including biannual IFV peaks, a rainy-season peak of RSV and HMPV, and year-round HRV circulation with a peak in July [4,5,24]. This pattern aligns with previous reports suggesting that the initial post–pandemic resurgence of respiratory viruses reflected a temporary increase in susceptible population, with most viruses gradually returning to their typical pre–pandemic seasonal patterns during the second resurgence, as observed for RSV following the 2009 influenza pandemic [7,25,26]. Similarly, country-level analysis has demonstrated marked post–pandemic shifts in seasonality, with epidemic durations subsequently returning to typical patterns, while overall activity remained low in 2024 [7]. Notably, SARS-CoV-2 activity increased between April and May (warm season), likely influenced by changes in human behavior, such as outdoor gatherings and crowding during the Songkran festival, and potentially driven by the emergence of new variants or subvariants [27]. In addition, previous studies have indicated that the activity of respiratory viruses was positively correlated with relative humidity and rainfall [5,24]. Therefore, it would be interesting to further assess the impact of local meteorological factors on shaping viral circulation in 2024.

Thailand, located in tropical Southeast Asia, shares similar climatic conditions with neighboring countries such as Myanmar, Cambodia, Laos PDR, and the Philippines. However, the influenza activity patterns in Thailand differ slightly from those of its neighbors [28]. While these neighboring countries generally exhibit a single distinct peak in influenza activity around September, coinciding with the rainy season [28,29,30], Thailand displays two distinct peaks, with the major peak also occurring during the rainy season. These observations suggest that local climatic factors likely play an important role in shaping variations in respiratory virus activity [5,24]. Although respiratory virus circulation in tropical regions is diverse, a global systematic analysis showed that countries located at similar latitudes and sharing comparable climatic conditions tend to exhibit comparable timing and duration of respiratory epidemics [4,28]. Therefore, countries lacking comprehensive surveillance data but sharing similar geographic and climatological conditions may benefit from understanding the seasonal patterns and characteristics of respiratory viruses circulating in comparable regions.

Children exhibit higher incidence associated with upper respiratory tract infections (URTIs) compared to adults and elderly populations [1]. Consistent with our findings, the overall positive detection rate for viral pathogens in 2024 was highest in children. Among children aged < 2 years, the three predominant viral pathogens were HRV, SARS-CoV-2, and RSV. HRV accounts for more than half of common cold cases and was not only the most frequently detected pathogen in children but also prevalent across all age groups, underscoring its crucial role in respiratory infections [31]. The high incidence of SARS-CoV-2 in children under 2 years of age aligns with our previous report, which showed that more than 80% of children in this age group were seropositive, indicating widespread exposure within the first two years of life [32]. Similarly, the high prevalence of RSV in children under two years of age is consistent with previous findings showing that maternally derived antibodies decline by approximately seven months of age, and that seroconversion occurs within the first two years of life, indicating natural infection [33]. Our findings showed that RSV prevalence among preschool children aged 3–5 years in 2024 did not reach the levels observed in earlier seasons. This may be explained by the rapid resurgence of RSV during 2022–2023, shortly after the lifting of NPIs, was largely driven by increasing social interactions among children and waning population immunity, a phenomenon often referred to as “immunity debt” [34]. Consequently, prior exposure RSV of most children in this age group likely reduced the pool of susceptible individuals, leading to a lower burden of cases in 2024.

Among school-age children (6–11 years), IFV, HRV, and HAdV were the most frequently detected pathogens. This pattern suggests that age-related factors, such as close contact in schools and nurseries and school reopening, can significantly influence viral transmission [9]. In adolescents, adults, and older adults, IFV and SARS-CoV-2 were the most frequently detected pathogens, consistent with previous reports showing higher influenza prevalence in adults compared to children [20].

The high prevalence of IFV and SARS-CoV-2 in adults and the elderly, particularly among high-risk groups, underscores the importance of vaccination in alleviating healthcare pressures, especially in low- and middle-income countries where severe disease is more common. Currently, no licensed vaccines are available for HRV, HPIV, or HMPV, although several candidates are in early-stage clinical evaluation [35,36]. For RSV, preventive strategies now include the use of the monoclonal antibody nirsevimab and vaccines based on adjuvanted prefusion F protein, which are approved for older adults and pregnant women [37,38]. Understanding the epidemiology and seasonal patterns of respiratory viruses is crucial for informing vaccine policy, as administering vaccines prior to seasonal peaks is likely to reduce the burden of respiratory infections.

This study has several limitations. First, the relatively short study period constrained our ability to fully characterize seasonal patterns, particularly for SARS-CoV-2 in the post–COVID-19 era. Longer-term surveillance across multiple seasons is required to gain a more comprehensive understanding of viral pathogen dynamics and to determine whether the observed seasonal patterns represent temporary shifts or sustained changes in peak activity. Second, the data were collected from a single sampling site, which may limit the representativeness of the findings for other geographic areas. Third, the absence of clinical severity indicators, such as hospitalization, ICU admission, or mortality data, limited our ability to assess disease burden and clinical outcomes. Forth, we did not investigate bacterial pathogens associated with ARIs. Previous studies have indicated that bacterial detections are less frequent than viral detections in ARIs, with *Mycoplasma pneumoniae* being the most commonly identified bacterium [8,14]. However, the lack of bacterial assessment in our study may have resulted in a lower overall co-infection rate compared with studies that included bacterial–bacterial or viral–bacterial co-infections [14,23]. Finally, other potential determinants, such as environmental factors, which may influence seasonality, were not assessed [4,5,6].

## 5. Conclusions

In conclusion, this study provides additional insights into the profiles of respiratory viral pathogens, patterns of co-infection, and the age- and season-specific characteristics of viral spread in patients with ARIs. These findings can inform healthcare strategies for managing potential surges, facilitating the implementation of targeted interventions and appropriate control measures during periods of high viral activity. Moreover, the results provide valuable guidance for the development of vaccine policies.

## Figures and Tables

**Table 1:** Demographic data of participants.

	Samples (%) (N = 7853)
Age, year (mean, SD)	28.1 (22.5)
Age group	
Infant (0–2 years)	705 (9)
Pre-school children (3–5 years)	971 (12.3)
School-age children (6–12 years)	1228 (15.6)
Adolescents (13–18 years)	399 (5.1)
Adults (19–60 years)	3789 (48.3)
Older adults (>60 years)	761 (9.7)
Gender	
Female	4309 (54.9)
Male	3544 (45.1)

## Data Availability

All data are available in this manuscript. Additional information can be obtained from the corresponding author upon reasonable request.

## Supplementary Materials

The following supporting information can be downloaded at: https://www.mdpi.com/article/10.3390/tropicalmed10120339/s1, Table S1: Primers and probes used to detect influenza virus (IFV), RSV, hMPV, HRV, HPIV, HAdV, HCoV and SARS-CoV-2.

## References

[1] Chen C., You Y., Du Y., Zhou W., Jiang D., Cao K., Yang M., Wu X., Chen M., Qi J. (2024). Global epidemiological trends in the incidence and deaths of acute respiratory infections from 1990 to 2021. Heliyon.

[2] Bender R.G., Sirota S.B., Swetschinski L.R., Dominguez R.M.V., Novotney A., Wool E.E., Ikuta K.S., Vongpradith A., Rogowski E.L.B., Doxey M. (2024). Global, regional, and national incidence and mortality burden of non-COVID-19 lower respiratory infections and aetiologies, 1990–2021: A systematic analysis from the Global Burden of Disease Study 2021. Lancet Infect. Dis..

[3] Kesson A.M. (2007). Respiratory virus infections. Paediatr. Respir. Rev..

[4] Li Y., Reeves R.M., Wang X., Bassat Q., Brooks W.A., Cohen C., Moore D.P., Nunes M., Rath B., Campbell H. (2019). Global patterns in monthly activity of influenza virus, respiratory syncytial virus, parainfluenza virus, and metapneumovirus: A systematic analysis. Lancet Glob. Health.

[5] Suntronwong N., Vichaiwattana P., Klinfueng S., Korkong S., Thongmee T., Vongpunsawad S., Poovorawan Y. (2020). Climate factors influence seasonal influenza activity in Bangkok, Thailand. PLoS ONE.

[6] Bull J.J., Koelle K., Antia R. (2025). Waning immunity drives respiratory virus evolution and reinfection. Evol. Med. Public Health.

[7] Chen X., Chen H., Tao F., Chen Y., Zhou Y., Cheng J., Wang X. (2025). Global analysis of influenza epidemic characteristics in the first two seasons after lifting the nonpharmaceutical interventions for COVID-19. Int. J. Infect. Dis..

[8] Zhao P., Zhang Y., Wang J., Li Y., Wang Y., Gao Y., Zhao M., Zhao M., Tan H., Tie Y. (2024). Epidemiology of respiratory pathogens in patients with acute respiratory infections during the COVID-19 pandemic and after easing of COVID-19 restrictions. Microbiol. Spectr..

[9] Chow E.J., Uyeki T.M., Chu H.Y. (2023). The effects of the COVID-19 pandemic on community respiratory virus activity. Nat. Rev. Microbiol..

[10] Suntronwong N., Thongpan I., Chuchaona W., Budi Lestari F., Vichaiwattana P., Yorsaeng R., Pasittungkul S., Kitphati R., Vongpunsawad S., Poovorawan Y. (2020). Impact of COVID-19 public health interventions on influenza incidence in Thailand. Pathog. Glob. Health.

[11] Del Riccio M., Caini S., Bonaccorsi G., Lorini C., Paget J., van der Velden K., Meijer A., Haag M., McGovern I., Zanobini P. (2024). Global analysis of respiratory viral circulation and timing of epidemics in the pre–COVID-19 and COVID-19 pandemic eras, based on data from the Global Influenza Surveillance and Response System (GISRS). Int. J. Infect. Dis..

[12] Lu C., Barr I.G., Lambert S., Mengersen K., Wang L., Yang W., Li Z., Vardoulakis S., Bambrick H., Hu W. (2025). Shifts in seasonal influenza patterns in Australia during and after COVID-19: A comprehensive analysis. J. Infect. Public Health.

[13] Li Y., Wang X., Blau D.M., Caballero M.T., Feikin D.R., Gill C.J., Madhi S.A., Omer S.B., Simões E.A.F., Campbell H. (2022). Global, regional, and national disease burden estimates of acute lower respiratory infections due to respiratory syncytial virus in children younger than 5 years in 2019: A systematic analysis. Lancet.

[14] Inma P., Suntronwong N., Sinsulpsiri S., Srimaneewiroon S., Poovorawan Y. (2024). Viral Etiology Associated with Acute Respiratory Tract Infection Patients in Bangkok, Thailand. Cureus.

[15] Centers for Disease Control and Prevention (2020). Research Uses Only 2019-Novel Coronavirus (2019-nCoV) Real-Time RT-PCR Primers and Probes. https://stacks.cdc.gov/view/cdc/88834.

[16] Gunson R., Collins T., Carman W. (2005). Real-time RT-PCR detection of 12 respiratory viral infections in four triplex reactions. J. Clin. Virol..

[17] Jiang X.W., Huang T.S., Xie L., Chen S.Z., Wang S.D., Huang Z.W., Li X.Y., Ling W.P. (2022). Development of a diagnostic assay by three-tube multiplex real-time PCR for simultaneous detection of nine microorganisms causing acute respiratory infections. Sci. Rep..

[18] Suwannakarn K., Payungporn S., Chieochansin T., Samransamruajkit R., Amonsin A., Songserm T., Chaisingh A., Chamnanpood P., Chutinimitkul S., Theamboonlers A. (2008). Typing (A/B) and subtyping (H1/H3/H5) of influenza A viruses by multiplex real-time RT-PCR assays. J. Virol. Methods.

[19] Thermofisher Parainfluenza Virus Research Using a Multiplex Real-Time RT-PCR Method and the ViiA™ 7 Real-Time PCR System. https://documents.thermofisher.com/TFS-Assets/LSG/brochures/cms_088565.pdf.

[20] Thongpan I., Suntronwong N., Vichaiwattana P., Wanlapakorn N., Vongpunsawad S., Poovorawan Y. (2019). Respiratory syncytial virus, human metapneumovirus, and influenza virus infection in Bangkok, 2016–2017. PeerJ.

[21] Zhao M., Xu Y., Zhang D., Li G., Gao H., Zeng X., Tie Y., Wu Y., Dai E., Feng Z. (2022). Establishment and evaluation of a quadruple quantitative real-time PCR assay for simultaneous detection of human coronavirus subtypes. Virol. J..

[22] World Health Organization Global Trends in Infectious Respiratory Diseases. https://apps.who.int/gb/MSPI/pdf_files/2024/12/Item1_19-12.pdf.

[23] Liu Y.N., Zhang Y.F., Xu Q., Qiu Y., Lu Q.B., Wang T., Zhang X.A., Lin S.H., Lv C.L., Jiang B.G. (2023). Infection and co-infection patterns of community-acquired pneumonia in patients of different ages in China from 2009 to 2020: A national surveillance study. Lancet Microbe.

[24] Thongpan I., Vongpunsawad S., Poovorawan Y. (2020). Respiratory syncytial virus infection trend is associated with meteorological factors. Sci. Rep..

[25] Li Y., Wang X., Msosa T., de Wit F., Murdock J., Nair H. (2021). The impact of the 2009 influenza pandemic on the seasonality of human respiratory syncytial virus: A systematic analysis. Influenza Other Respir. Viruses.

[26] Zhao C., Zhang T., Guo L., Sun S., Miao Y., Yung C.F., Tomlinson J., Stolyarov K., Shchomak Z., Poovorawan Y. (2025). Characterising the asynchronous resurgence of common respiratory viruses following the COVID-19 pandemic. Nat. Commun..

[27] World Health Organization (2025). WHO COVID-19 Dashboard. https://data.who.int/dashboards/covid19/variants.

[28] Hirve S., Newman L.P., Paget J., Azziz-Baumgartner E., Fitzner J., Bhat N., Vandemaele K., Zhang W. (2016). Influenza seasonality in the tropics and subtropics—When to vaccinate?. PLoS ONE.

[29] Horwood P.F., Karlsson E.A., Horm S.V., Ly S., Heng S., Chin S., Darapheak C., Saunders D., Chanthap L., Rith S. (2019). Circulation and characterization of seasonal influenza viruses in Cambodia, 2012–2015. Influenza Other Respir. Viruses.

[30] Wodniak N., Vilivong K., Khamphaphongphane B., Sengkeopraseuth B., Somoulay V., Chiew M., Ketmayoon P., Jiao M., Phimmasine S., Co K.C. (2024). Epidemiologic and Virologic Characteristics of Influenza in Lao PDR, 2016–2023. Influenza Other Respir. Viruses.

[31] Jacobs S.E., Lamson D.M., St George K., Walsh T.J. (2013). Human rhinoviruses. Clin. Microbiol. Rev..

[32] Suntronwong N., Assawakosri S., Klinfueng S., Duangchinda T., Chantima W., Pakchotanon P., Nilyanimit P., Vichaiwattana P., Aeemjinda R., Wongsrisang L. (2025). Age-associated SARS-CoV-2 immune responses provide insights into population immunity over four years since the COVID-19 pandemic. Sci. Rep..

[33] Pasittungkul S., Thongpan I., Vichaiwattana P., Thongmee T., Klinfueng S., Suntronwong N., Wanlapakorn N., Vongpunsawad S., Poovorawan Y. (2022). High seroprevalence of antibodies against human respiratory syncytial virus and evidence of respiratory syncytial virus reinfection in young children in Thailand. Int. J. Infect. Dis..

[34] Jiang W., Xu L., Wang Y., Hao C. (2024). Exploring immunity debt: Dynamic alterations in RSV antibody levels in children under 5 years during the COVID-19 pandemic. J. Infect..

[35] Stobart C., Nosek J., Moore M. (2017). Rhinovirus biology, antigenic diversity, and advancements in the design of a human rhinovirus vaccine. Front. Microbiol..

[36] Schnyder Ghamloush S., Essink B., Hu B., Kalidindi S., Morsy L., Egwuenu-Dumbuya C., Kapoor A., Girard B., Dhar R., Lackey R. (2024). Safety and immunogenicity of an mRNA-based HMPV/PIV3 combination vaccine in seropositive children. Pediatrics.

[37] Centers for Disease Control and Prevention Vaccines and Immunizations. https://www.cdc.gov/vaccines/vpd/rsv/hcp/older-adults.html.

[38] Jones J.M. (2023). Use of nirsevimab for the prevention of respiratory syncytial virus disease among infants and young children: Recommendations of the Advisory Committee on Immunization Practices—United States, 2023. MMWR Morb. Mortal. Wkly. Rep..

