# Supplementary material for: Seasonal Pattern and Age-Specific Detection of Eight Respiratory Viruses Causing Acute Respiratory Infection in 2024, Bangkok, Thailand
Source: Trop Med Infect Dis. 2025 Nov 29;10(12):339. doi: 10.3390/tropicalmed10120339 (PMC12737683; doi:10.3390/tropicalmed10120339)
Supplement: Supplementary file 1 [file tropicalmed-10-00339-s001.zip › tropicalmed-3935129-supplementary.pdf]

Supplementary Table S1. Primers and probes used to detect influenza virus (IFV), RSV, hMPV, HRV, HPIV, HAdV, HCoV and SARS-CoV-2.

	Virus	Primer/Probe name	Nucleotide sequence 5'->3'	Target gene	Reference
Assay 1	GAPDH	GAPDH-F	GTGAAGGTCGGAGTSAACGG	GAPDH	[1]
		GAPDH-R	TCAATGAAGGGGTCATTGATGG	GAPDH	
		GAPDH-P (Probe)	HEX-CGCCTGGTCACCAGGGCTGC-BHQ1	GAPDH	
	Influenza A	FluA-M-F151	CATGGARTGGCTAAAGACAAGACC	M	
		FluA-M-R276	AGGGCATTTTGGACAAAKCGTCTA	M	
		FluA-M-P218	FAM-ACGCTCACCGTGCCCAGT-BHQ1	M	
	Influenza B	FluB-MF439	CTCTGTGCTTTRTGCGARAAAC	M	
		FluB-MR	CCTTCYCCATTCTTTTGACTTGC	M	
		FluB-P135	Cy5-TCAGCAATGAACACAGCAA-BHQ3	M	
Assay 2	RSV	RSV_F3251	GGCAAATATGGAAACATACGTGAA	M	[2]
		RSV_R3334	TCTTTTTCTAGGACATTGTAYTGAACAG	M	
		RSV_P3303	FAM-CTGTGTATGTGGAGCCTTCGTGAAGCT-BHQ1	M	
	hMPV	hMPV_F3604	CAARTGYGACATTGCTGAYCTRAA	F	
		hMPV_R3683	ACTGCCGCACAACATTTAGRAA	F	
		hMPV_P3630	JOE-TGGCYGTYAGCTTCAGTCARTTC-BHQ1	F	
Assay 3	HRV	qPCR/HRV_F	GACAWGGTGTGAAGAGYC	5'UTR	[3]
		qPCR/HRV_R	CAAAGTAGTYGGTYCCRTCC	5'UTR	
		qPCR/HRV_P	FAM-TCCTCCGGCCCCTGAAT-3IABkFQ	5'UTR	
Assay 4	HPIV1	PIV1_F2	AAAAACTTAGGGTTAAAGACAATCCA	HN	[4]
		PIV1_R2_N	GCCAGATGTRTRTCCTTCCTGCTGGT	HN	
		PIV1-P-N (Probe)	HEX-AAACGATGGCTGAAAAAGGGAA-BHQ1	HN	
	HPIV2	PIV2_F2_N	CCATYTACCTAAGTGATGGAA	HN	
		PIV2_R_N	TGGCATAATCTTCTTTYTCAG	HN	
		PIV2-P (Probe)	FAM-AATCGCAAAAGCTGTTCAGTCAC-BHQ1	HN	
	HPIV3	PIV3_F2	CAGGAAGCATTGTRTCATCTGT	HN	
		PIV3_R2_N	ATAGTGTGTAATGCAGCTTGT	HN	
		PIV3-P (Probe)	FAM-ACCCAGTCATAACTTACTCAACAGCAAC-BHQ1	HN	
	HPIV4	PIV4_F1	CAAAYGATCCACAGCAAAGATTC	N	
		PIV4_R_N	ATGTGGCCTGYAARGAAAGCA	N	
		PIV4_P_N (Probe)	HEX-GTATCATCATCTGCCAAATCRGCAATTAAACA-BHQ1	N	
Assay 5	HAdV	qPCR/HAdV_F	GTAGACTTGCARGACAGAAACAC	Hexon	[5]
		qPCR/HAdV_R1	AAYRCGAACATCRGGATCRTAAC	Hexon	
		qPCR/HAdV_R2	TGATTCTAACATCDGGATCATAGC	Hexon	
		qPCR/HAdV_Probe	5'FAM-ATGTGGAABCAGGCTGTTGAC-3'MGB-NFQ	Hexon	
Assay 6	HCoV-229E	qPCR/229E_F	ACTTTGTCAGTTCGTATGCTAAAC	ORF1ab	[6]
		qPCR/229E_R	TTGTCAGAACATTGGCATTAACA	ORF1ab	
		qPCR/229E_Probe	FAM-ATTATCAGCTTATGACTTGGCGTGT-BHQ1	ORF1ab	
	HCoV-OC43	qPCR/OC43_F	ATTATCAGCTTATGACTTGGCGTGT	ORF1ab	

		qPCR/OC43_R	TCTGGACCACTATTAACAACCTG	ORF1ab	
		qPCR/OC43_Probe	HEX-TTGCAAAATAATGAATTAATGCCTGCTAAGTTGA-BHQ1	ORF1ab	
	HCoV-NL63	qPCR/NL63_F	GTTCTCTTATAGGTGGCATGGT	ORF1ab	
		qPCR/NL63_R	GAAGCACATCAGTTTGTAAAGCA	ORF1ab	
		qPCR/NL63_Probe	HEX-CCTTTTTCTTTGGCACTGCAAGCAC-BHQ1	ORF1ab	
	HCoV-HKU1	qPCR/HKU1_F	AGTTTATCTTTAGTTGATGTTTGGGA	ORF1ab	
		qPCR/HKU1_R	GATTTAACTAGGCGTGACAATTCAT	ORF1ab	
		qPCR/HKU1_Probe	FAM-TATTTGACAGGTTGTGATTATGTTGTTTGGG-BHQ1	ORF1ab	
Assay 7	SARS-CoV-2	N1_F 28286-28305	GACCCCAAAATCAGCGAAAT	N	[7]
		N1_R 28334-28357	TCTGGTTACTGCCAGTTGAATCTG	N	
		N1_Probe	FAM-ACC CCG CAT TAC GTT TGG TGG ACC-BHQ1	N	
		N2_F 29163-29182	TTA CAA ACA TTG GCC GCA AA	N	
		N2_R 29212-29229	GCG CGA CAT TCC GAA GAA	N	
		N2_Probe	FAM-ACA ATT TGC CCC CAG CGC TTC AG-BHQ1	N	

Abbreviations: GAPDH; glyceraldehyde 3-phosphate dehydrogenase, M; matrix gene, F; fusion gene, 5'UTR; 5′ untranslated region, N; nucleocapsid gene, HN; hemagglutinin-neuraminidase gene

Reference:

1. Suwannakarn, K., et al., Typing (A/B) and subtyping (H1/H3/H5) of influenza A viruses by multiplex real-time RT-PCR assays. Journal of virological methods, 2008. **152**(1-2): p. 25-31.
2. Thongpan, I., et al., Respiratory syncytial virus, human metapneumovirus, and influenza virus infection in Bangkok, 2016-2017. PeerJ, 2019. **7**: p. e6748.
3. Gunson, R., T. Collins, and W. Carman, Real-time RT-PCR detection of 12 respiratory viral infections in four triplex reactions. Journal of clinical virology, 2005. **33**(4): p. 341-344.
4. Thermofisher, Parainfluenza virus research using a Multiplex real-time rt-Pcr Method and the ViiA™ 7 Real-Time PCR system. Available online: https://documents.thermofisher.com/TFS-Assets/LSG/brochures/cms_088565.pdf (accessed on 17 September 2025).
5. Jiang, X.-W., et al., Development of a diagnostic assay by three-tube multiplex real-time PCR for simultaneous detection of nine microorganisms causing acute respiratory infections. Scientific Reports, 2022. **12**(1): p. 13306.
6. Zhao, M., et al., Establishment and evaluation of a quadruple quantitative real-time PCR assay for simultaneous detection of human coronavirus subtypes. Virology Journal, 2022. **19**(1): p. 67.
7. Centers for Disease Control and Prevention. Research use only 2019-novel coronavirus (2019-nCoV) real-time RT-PCR primers and probes. 2020. Available online: https://stacks.cdc.gov/view/cdc/88834 (accessed on 17 May 2025).
